# Human Papillomavirus Related Neoplasia of the Ocular Adnexa

**DOI:** 10.3390/v13081522

**Published:** 2021-08-02

**Authors:** Ingvild Ramberg, Steffen Heegaard

**Affiliations:** 1 Department of Pathology, Copenhagen University Hospital Rigshospitalet, DK-2100 Copenhagen, Denmark; Ingvild.margrethe.sellaeg.ramberg@regionh.dk; 2 Department of Ophthalmology, Copenhagen University Hospital Rigshospitalet, DK-2100 Copenhagen, Denmark

**Keywords:** human papillomavirus, ocular adnexa, conjunctiva, lacrimal drainage system, eyelids, squamous cell carcinoma, squamous cell papilloma, sebaceous cell carcinoma

## Abstract

Human papillomaviruses (HPV) are a large group of DNA viruses that infect the basal cells of the stratified epithelium at different anatomic locations. In the ocular adnexal region, the mucosa of the conjunctiva and the lacrimal drainage system, as well as the eyelid skin, are potential locations for HPV-related neoplasia. The role of HPV in squamous cell neoplasia of the ocular adnexa has been debated for several decades. Due to the rarity of all these tumors, large studies are not available in the scientific literature, thereby hampering the precision of the HPV prevalence estimates and the ability to conclude. Nevertheless, increasing evidence supports that defined subsets of conjunctival papillomas, intraepithelial neoplasia, and carcinomas develop in an HPV-dependent pathway. The role of HPV in squamous cell tumors arising in the lacrimal drainage system and the eyelid is still uncertain. Further, the potential of HPV status as a diagnostic, prognostic, or predictive biomarker in these diseases is a topic for future research.

## 1. Introduction

Human papillomaviruses (HPV) are a large group of DNA viruses with tropism for epithelial tissues of the skin and mucosae. Tremendous progress has been made within the field since the first report of a cell-free transmission of canine oral warts in 1898, and we are today aware of the substantial burden of neoplastic diseases caused by papillomaviruses in many anatomical locations [1]. HPV belongs to the *Papillomaviridae* family and is divided into five different genera (α, β, γ, μ, and *ν*) based on the homology of the nucleotide sequence coding for the HPV capsid protein L1. Most mucosal genotypes belong to the α-genus, whereas cutaneous genotypes predominantly belong to the α, β-, and γ-genera. Thus, in the ocular adnexa, the mucous membranes of the conjunctiva and the lacrimal drainage system, as well as the eyelid, are potential locations for HPV-related neoplasia.

Generally, studies investigating the association between HPV and neoplasia of the ocular adnexa are hampered by small sample sizes leading to imprecise HPV prevalence estimates. The studies are mostly limited to retrospective case series, and thereby inducing inherent risks of publication and selection biases. In addition, a great diversity of applied methods for HPV detection, with different sensitivity and specificity profiles, has been applied as described by Stagner et al. [2]. With these precautions, the role of HPV in neoplasia in the ocular adnexa is discussed in the present review.

## 2. The Conjunctiva

**Figure 1:**
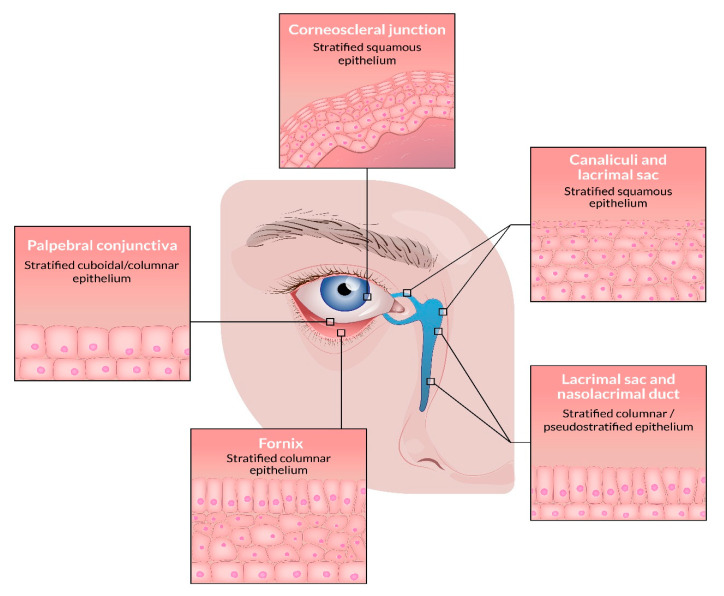
The epithelial linings of the conjunctiva and lacrimal drainage system.

The conjunctiva is the transparent mucous membrane covering the inside of the eyelids (palpebral conjunctiva) and the anterior part of the sclera (bulbar conjunctiva), where it becomes continuous with the anterior epithelium of the cornea (Figure 1). A non-keratinizing stratified columnar epithelium covers the palpebral conjunctiva that gradually thickens toward the fornices, and at the corneoscleral junction, the epithelium becomes squamous. Being the only mucous membrane in the body exposed directly to the external environment, the conjunctiva is vulnerable to external stress stimuli, including pathogens, ultraviolet (UV) radiation, air, dust, and cigarette smoke. The role of HPV in the pathogenesis of conjunctival neoplasia has been debated in the scientific literature since the first reports of HPV in conjunctival papillomas in 1983 [3] and conjunctival dysplasia and carcinoma in 1986 [4].

### 2.1. Conjunctival Papilloma

Conjunctival papilloma is a benign tumor arising from the conjunctival epithelium. Based on the growth pattern, the papillomas are histopathologically divided into exophytic and inverted papilloma. Rarely, a mixed growth pattern is present. The *exophytic papilloma* forms outwards projections of an acanthotic conjunctival epithelium surrounding a fibrovascular core. The papillomas can be sessile or pedunculated. Mild to moderate dysplastic changes may be present; however, severe dysplasia and malignant transformation are rarely seen [5,6,7]. Precise incidence estimates of conjunctival papillomas are not available in the scientific literature, but the disease accounts for 1–16% of all histopathologically verified conjunctival lesions [8]. The incidence peaks in patients aged 21–40 years and thereafter declines with a male predominance in all age groups [5,6,8]. 

Human papillomavirus is considered the main risk factor for developing these papillomas, consistently reported in more than 50% of the cases (Table 1) and with a reported viral load comparable to the levels in laryngeal papillomatosis [9]. The vast majority of detected genotypes are the low-risk HPV6 and HPV11, and rarely other genotypes such as HPV13, 16, 33, and 45 [10,11,12,13]. Histopathologically, the presence of basaloid cells, intraepithelial goblet cells, a non-keratinizing squamous epithelium, and lack of solar elastosis are associated with HPV [9]. On the other hand, koilocytosis is not a valid biomarker for HPV-related conjunctival papilloma [8,11,14]. Clinically, a location of a papilloma other than at the corneal limbus is associated with HPV infection [9]. A prognostic and predictive value of HPV status in conjunctival papilloma is not yet determined.

Opposed to the exophytic papillomas, the *inverted conjunctival papillomas* grow inwards, however, respecting the basement membrane, which remains intact. Inverted conjunctival papillomas are rare, with only a handful reported in the scientific literature to date [19]. An association with high-risk HPV16 and 58 genotypes has been reported [7,19,20]. Despite the limited reports, the inverted papillomas seem more aggressive than their exophytic counterparts with frequent synchronous and metachronous cancer development [7,20,21,22]. Although some publication bias is expected, this picture suits well with our knowledge from the adjacent sinonasal tract, where inverted papillomas often are associated with high-risk HPV genotypes and carry a significant risk of malignant transformation [23]. Thereby, close attention is required in the follow-up of these patients.

### 2.2. Conjunctival Intraepithelial Neoplasia and Squamous Cell Carcinoma

**Figure 2:**
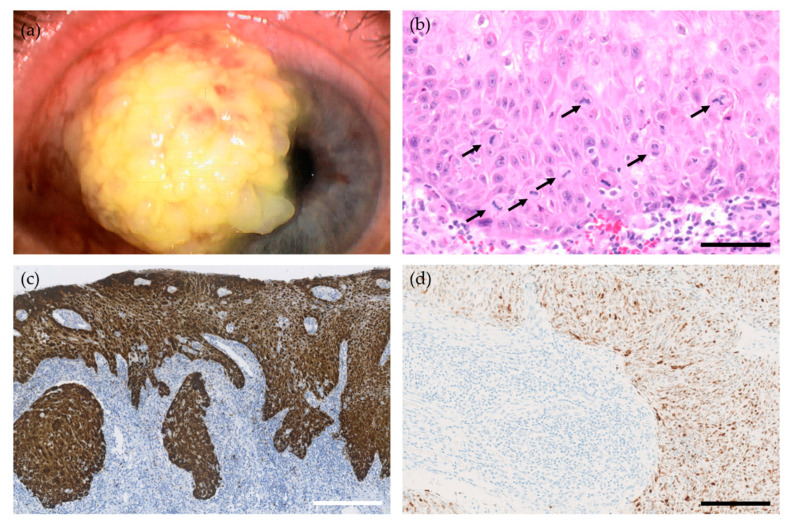
(**a**) A limbal conjunctival squamous cell carcinoma in situ with corneal involvement. (**b**) Histology reveals pleomorphic squamous tumor cells with abundant, aberrant mitoses (arrows) and subepithelial inflammation (hematoxylin-eosin (HE)-stain, bar scale = 100 μm). (**c**) Intense positive cytoplasmatic and nuclear expression of p16^INK4a^ in the tumor cells, a surrogate marker of human papillomavirus (HPV) infection (p16^INK4a^, immunohistochemistry, scale bar = 450 μm). (**d**) Expression of high-risk HPV oncogenes within the tumor cells. Note the clear demarcation to the underlying stromal tissue (HPV E6/E7 mRNA in-situ hybridization using a probe for detection of high-risk HPV genotypes (16, 18, 26, 31, 33, 35, 39, 45, 51, 52, 53, 56, 58, 59, 66, 68, 73, and 82), scale bar = 350 μm).

Conjunctival squamous cell carcinoma (SCC) is the most common malignancy arising from the conjunctiva and is the end-stage disease in the spectrum ranging from conjunctival intraepithelial neoplasia (CIN) grades I-III, carcinoma in-situ, and eventually invasive conjunctival SCC (Figure 2). While CIN encompass different degrees of epithelial involvement only, the conjunctival SCC invades the basement membrane and the underlying substantia propria. Conjunctival SCC has a locally destructive growth pattern, where the malignant cells eventually invade the adjacent structures, including the globe (American Joint Committee on Cancer (AJCC) tumor stage T3) and the orbit (AJCC tumor stage T4). Orbital exenteration is required in approximately 10% of the cases to obtain local tumor control [24,25] causing significant morbidity. However, also AJCC tumor stage T1-2 disease can cause vision loss due to limbal stem cell deficiency. Conjunctival SCC also carries a risk of lymph node- and systemic metastasis, especially in people living with human immunodeficiency virus (HIV)—a patient group that evolves more frequent and more severe disease at a younger age [26,27,28,29].

#### 2.2.1. Prognosis and Treatment

One of the main challenges in treating conjunctival SCC is the high recurrence rates [30]. A systematic review of the literature reported a mean risk of recurrence at 52% in cases with positive surgical margins and 11% in cases with negative surgical margins [31]. Adjuvant therapy, including topical or intralesional chemotherapy, radiotherapy, or plaque brachytherapy, further reduces the persistence of the tumors and the recurrence rates, independent of margin status, but may also cause considerable side effects [29,32]. The application of adjuvant topical interferon alpha-2b in cases with narrow, indeterminate, or negative surgical margins seems to obtain better tumor control than re-excision, incisional biopsy-guided interferon alpha-2b therapy, or empiric interferon alpha-2b treatment as monotherapy [31]. Still, non-responsiveness to interferon treatment is relatively common, with a reported pooled estimate of 12% of the cases reported in a systematic literature review [31,33]. Moreover, in a randomized controlled study, monotherapy with mitomycin C in conjunctival CIN could not be separated histologically from the placebo group after 6–8 weeks of treatment [34].

The high recurrence rates and resistance to treatment are clinical challenges, as the risk of adverse events, including corneal and conjunctival scarring, corneal stem cell deficiency, and risk of orbital exenteration, increases by repeated intervention. Therefore, in order to up- and de-escalate the treatment, also in pre-invasive tumors, there is an urge for randomized controlled studies, identification of subgroups, and incorporation of valid biomarkers.

#### 2.2.2. Human Papillomavirus in Conjunctival CIN and SCC

As mentioned, the clinical course of HIV-associated conjunctival SCC differs substantially from those arising in immunocompetent individuals, and HIV status delineates two important subgroups to be considered in future trials. Increasing evidence also supports the role of HPV in conjunctival CIN and SCC, and HPV status may also serve as an important biomarker in these diseases. With an odds ratio (OR) 8.42 (95%CI 3.70–19.16 in 21 studies) [35] and a risk ratio (RR) 3.13 (95%CI 1.72–5.71, 16 studies) [36] in two separate meta-analyses based on observational studies, HPV has shown to be strongly associated with an increased risk of conjunctival CIN and SCC. The prevalence estimates of HPV are 26% (95%CI 13–42%) in conjunctival CIN and 18% (95%CI 7–32%) in invasive SCC compared to 1% (95%CI 0–3%) among controls [35]. An overview of studies examining HPV in conjunctival intraepithelial neoplasia and carcinoma (39 studies) is provided in a recently systematic review by our group [35].

The expression of the viral oncogenes E6 and E7 within the tumor cells, and not merely detection of viral DNA by PCR, gives additional weight to a causative role of HPV in a subgroup of conjunctival CIN and SCC (Table 2) [33,37,38,39]. Further, the loss of HPV E4 gene expression in the malignant cells, hence a transition from a productive to an abortive life-cycle of the HPV, is documented in a series of conjunctival CIS similar to high-risk cervical intraepithelial neoplasia [33]. The high-risk genotype HPV16 is by far the most frequent genotype in conjunctival CIN and SCC, followed by HPV18 and HPV33 [35]. Less frequent, HPV6, 11, 31, 35, 37, 39, 44, 45, 51, 52, 58, 66, 72, and 83, are detected [35].

With stratification by geographical location, studies from African countries show an insignificant association to α HPV genotypes (OR 1.7, 95%CI 0.9–3.5) [35], but a significant association to β genus HPV genotypes (RR 3.52, 95%CI 1.23–10.08) [36]. The development of cancers caused by the β genus HPV is primarily seen in individuals with compromised immune surveillance, e.g., patients in systemic immunosuppressive treatment or people living with HIV [40]. The risk of conjunctival SCC is more than 10-fold in people living with HIV and is considered an early indicator of HIV infection [28]. The link between β genus HPV genotypes and conjunctival SCC in African countries may therefore be explained by the strong association between HIV and conjunctival SCC development [41], but is outside the scope of the present review.

#### 2.2.3. Clinical Characteristics of HPV-Related Conjunctival CIN and SCC

Only a few studies have compared the clinical characteristics of HPV-positive versus HPV-negative conjunctival CIN and SCC. Due to the retrospective setup of these studies, no conclusions regarding clinical outcomes of HPV status can be drawn on this basis. Our current knowledge suggests that HPV-positive conjunctival SCC develops at an earlier age than HPV-negative conjunctival SCC [38,42] with a higher risk of extraconjunctival extension at diagnosis [37] and a higher risk of recurrence [38]. One study has investigated the prognostic value of p16^INK4a^ in conjunctival SCC and found a significant reduced disease-free survival in patients with p16^INK4a^ -positive tumors. However, as HPV status was not reported in this study, this finding might be unrelated to HPV [43].

#### 2.2.4. Histological and Immunohistological Markers of HPV-Related Conjunctival CIN and SCC

Histologically, HPV-related conjunctival CIN and SCC are associated with a basaloid, non-keratinizing morphology with foci of comedo necrosis and a dense inflammatory background [38,42]. The presence of koilocytosis, hence cellular and nuclear enlargement, hyperchromatic nuclei, and a perinuclear cytoplasmic halo, is not a valid marker of HPV-related conjunctival CIN and SCC, with reported sensitivity ranging from 0 to 100% [17,44,45] and specificity ranging from 63 to 100% [17,44,46].

Evaluated by immunohistochemistry, p16^INK4a^ expression is a surrogate marker of HPV-related carcinomas in the head-and-neck region as well as anogenital carcinomas. Under homeostatic conditions, the p16 ^INK4a^ acts as a tumor suppressor by inactivating the cyclin-dependent kinases that phosphorylate the retinoblastoma protein (pRb) and thereby hamper the cell cycle progression from G1 to the S phase of mitosis. Phosphorylation of pRb in turn, influences the expression of p16^INK4a^. The HPV oncogene E7 deregulates pRB leading to a compensatory increase in p16^INK4a^ expression. The expression of p16^INK4a^ in conjunctival CIN and SCC has been evaluated in several studies [11,33,38,42,45,47,48,49,50,51,52]. However, no conclusions on the applicability of p16^INK4a^ in conjunctival CIN and SCC can be taken due to the broad variability in the definition of p16^INK4a^ overexpression (percentage of tumor cells or binary definition (positive/negative), nuclear and/or cytoplasmatic staining), the immunohistochemistry staining probes used, HPV results, and HPV detection modalities used (PCR-based (HPV DNA PCR, HPV RNA RT-PCR) and/or in-situ hybridization (ISH)-based (HPV DNA ISH, HPV RNA ISH) or combination of techniques). Two studies reported p16^INK4a^ IHC along with HPV DNA PCR and E6/E7 mRNA ISH [33,38]. In these two studies, the p16^INK4a^ IHC yielded a sensitivity ranging from 85 to 90% and a specificity ranging from 66 to 90% for a prediction of high-risk HPV infection when using HPV DNA PCR as a reference, and a sensitivity of 100% and a specificity 75% of when using mRNA ISH as a reference [33,38]. Hence, the value of p16^INK4a^ overexpression as a biomarker for HPV-related conjunctival CIN and SCC is a topic for future research.

### 2.3. Transmission of HPV to the Conjunctiva

There are several hypotheses of how HPV reaches the conjunctiva, and most likely, there are different transmittal routes. Looking at the genotypes involved in conjunctival pathogenesis, the low-risk HPV6 (“genital type”) and HPV11 (“oral type”) predominates in the exophytic conjunctival papillomas, whereas the high-risk HPV16 constitutes the vast majority in conjunctival SCC. Likely, the HPV is transferred from the anogenital region as well as the oral and pharyngeal mucosa to the conjunctiva. Vertical transmission—hence, acquired infection during the passage of an infected birth canal—may cause neonatal or early conjunctival papillomatosis [53,54,55]. In a prospective cohort study of HPV-positive pregnant women, HPV was detected in the conjunctiva in 4.8% of the children at birth and/or at three months of age [56]. Auto-inoculation is another proposed transmission mode, e.g., by hand carriage of genital HPV DNA [57]. Reports of co-existent genital- and conjunctival HPV-related neoplasia support autoinoculation as a transmission route to the conjunctiva [55,58].

For the virus to infect the basal cell of the epithelium, micro-abrasions of the conjunctival surface in order to expose the basal membrane are required. Such abrasions are common in patients with vitamin A deficiency and atopy—both patient groups with increased risk of conjunctival SCC [33,59]. Micro abrasions of the conjunctiva also occur in patients with ocular prostheses and thereby making the conjunctiva susceptible to HPV infection [60].

In conclusion, the conjunctiva is a vulnerable epithelial site of HPV-related neoplasia with viral and cellular biomarkers of deregulated HPV gene expression. However, increasing evidence also supports a diagnostic and prognostic value of high-risk HPV expression in conjunctival CIN and SCC.

## 3. The Lacrimal Drainage System

The lacrimal drainage system (LDS) drains tears, debris, and microbes from the ocular surface to the nasal cavity. Anatomically, the LDS consists of the lacrimal canaliculi, the lacrimal sac, and the nasolacrimal duct (Figure 1). Tumors arising in the LDS are often of epithelial origin, with the vast majority being squamous cell carcinoma and non-keratinizing squamous cell carcinoma (previously transitional cell carcinoma) [61]. Of benign tumors, squamous- and transitional papillomas occur most frequently. The papillomas of the LDS can be inverted, squamous/exophytic, transitional cell, or mixed type. The malignant tumors are most often located in the lacrimal sac and the nasolacrimal duct, whereas the benign tumors more often appear in the upper part of the LDS [62].

Tumors of the LDS are rare; hence, our current knowledge of these tumors’ development is scarce. Suspected risk factors for developing LDS carcinoma include chronic inflammation, infection with Epstein-Barr virus, previous probing or surgery of the LDS, and HPV infection [61]. Both exophytic and inverted papillomas of the LDS can undergo malignant transformation. Thereby, squamous cell carcinomas of the LDS can develop de novo or in a pre-existing papilloma [63,64,65]. The role of HPV in LDS papilloma and carcinoma development is still uncertain due to the rarity of the tumors. In LDS papillomas, mostly low-risk genotypes (HPV6, 11) have been reported; however, they are limited to a handful of case reports and small case series (Table 3). One study reports the expression of HPV oncogenes in the tumor cells, suggesting that HPV is not merely an innocent bystander in the development of these tumors’ development [66].

Regarding LDS carcinomas, only four studies in the literature have addressed the association to HPV (Table 4) [42,66,67,68]. In these studies, HPV was detected in 15 out of 25 (60%) squamous cell - and transitional cell carcinomas by HPV DNA PCR, most frequently being HPV16. However, as HPV DNA detection by PCR does not prove causality, further investigations are required to decide the role of HPV in LDS carcinoma development.

The treatment options of LDS carcinomas differ according to the histopathological diagnosis, the tumor location, and the stage of the disease. Due to the rarity of the diseases, the treatment regimens are often extrapolated from other head-and-neck cancers, especially sinonasal carcinomas. Surgical resection is the first-line treatment, often adjuvanted by intensity-modulated radiotherapy [71]. The benefits of adjuvant chemotherapy in the treatment of LDS carcinomas are still uncertain. Cisplatin-based chemotherapy is frequently used in patients with inoperable tumors and metastatic disease.

## 4. The Eyelids

The eyelids are covered by a keratinized, stratified squamous epithelium only a few cells thick. The main functions of the eyelids are to protect the globe and secrete, distribute, and drain tears on the ocular surface. The sebaceous glands of the eyelids (the Meibomian glands, the glands of Zeis, and the glands associated with the caruncle) are a part of the epidermal appendages and are responsible for the secretion of the oily part of the tear film and cilia. The reports of HPV-associated carcinomas deriving from the skin and the sebaceous glands of the ocular adnexa are discussed in the following sections.

### 4.1. Sebaceous Gland Carcinoma

**Figure 3:**
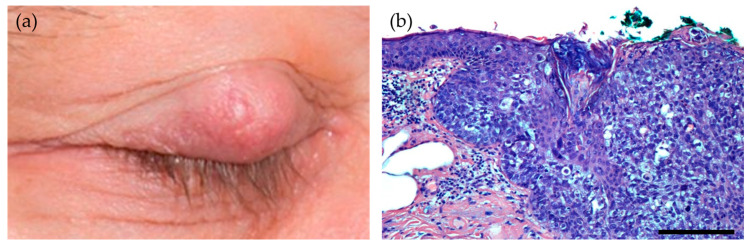
(**a**) A sebaceous gland carcinoma of the eyelid with origin in a meibomian gland. The clinical examination revealed a painless, firm papule of the upper eyelid. (**b**) Histopathologically, the tumor consists of pleomorphic sebaceous tumor cells with scattered mitoses and display pagetoid growth. The tumor is dominated by basaloid cells with only a few well-differentiated multivacuolated sebaceous tumor cells (hematoxylin-eosin (HE)-stain, scale bar = 175 μm).

Carcinomas of the sebaceous glands in the ocular adnexal skin structures most commonly arise in the Meibomian glands (Figure 3). Geography impacts the incidence, being more common in Asians (one-third of all eyelid malignancies) and rare in Caucasians (0.5–5% of all eyelid malignancies) [72].

Risk factors for the development of sebaceous carcinoma include UV-radiation, genetic predisposition syndromes (e.g., Muir-Torré syndrome), immunosuppression, previous radiation therapy to the head or neck, and, possibly, HPV. The genomic profile of ocular adnexal sebaceous carcinoma differs from the UV-induced and microsatellite instability (MSI) profiles that characterize sebaceous carcinoma of other anatomic locations [73]. Thereby, sebaceous gland carcinoma of the ocular adnexa represents a defined subset of sebaceous carcinomas [73].

#### Human Papillomavirus in Ocular Adnexal Sebaceous Carcinoma

Conflicting evidence exists regarding the role of HPV in ocular adnexal sebaceous carcinoma (Table 5). The highest HPV prevalence is reported in a study by Hayashi et al. using DNA ISH reporting HPV positivity in 13 out of 21 (62%) samples [72]. However, the HPV DNA signals were also present in the surrounding normal sebaceous glands and epidermis, causing doubt on the specificity of the applied method. Several other studies failed to detect HPV in their series [74,75,76]. Stagner et al. reported HPV16 in one out of 24 samples using PCR but did not detect viral expression by mRNA ISH [2], further questioning the role of HPV [72]. On the other hand, recent studies by Tetzlaff et al. [77] and Moore et al. [78] detected expression of HPV oncogenes restricted to the tumor cells in 14% and 18% of their series, respectively. Tetzlaff et al. further reported mutually exclusive genetic profiles according to HPV-status; *RB1/TP53* wildtype tumors harboring HPV as one group distinct to *RB1/TP53* co-mutant tumors without association to HPV [77]. The high frequency of *RB1* mutations in ocular adnexal sebaceous carcinoma leads to a compensatory increase in p16^INK4a^ expression. Hence, p16^INK4a^ expression is not a valuable surrogate marker of HPV infection in ocular adnexal sebaceous carcinoma [2,74].

Overall, the small sample sizes of the studies and the low prevalence of HPV in ocular adnexal sebaceous carcinoma lead to broad variations in the reported HPV prevalence. Nevertheless, the detection of transcriptionally active HPV within the tumor cells is a strong predictor of HPV involvement in a subset of ocular adnexal sebaceous carcinoma.

### 4.2. Cutaneous Squamous Cell Carcinoma

Cutaneous squamous cell carcinoma (SCC) is the second most common eyelid malignancy after basal cell carcinoma comprising approximately 10% of all eyelid malignancies [80,81]. Exposure to ultraviolet (UV) radiation and immunosuppression are the main risk factors for developing the disease. Moreover, β genus HPV types are suspected to promote cutaneous SCC formation, especially in immunocompromised patients [82,83,84]. Synergistically, the β genus HPV types are thought to interact with UV-radiation-induced genomic aberrations to promote cancer development [84]. There are several factors that question causality between HPV and cutaneous SCC. Most importantly, many HPV genotypes with cutaneous tropism are considered commensal viruses, hence a part of the human virome, causing asymptomatic infections rather than neoplastic disease [85]. Moreover, it seems as though β genus HPV is not required for cutaneous SCC maintenance and growth—different from the “classic” α-genus induced HPV-carcinogenesis [84]. Hence, the presence of HPV in apparently healthy skin, and the lack of viral expression in late stages of the disease questions the importance of HPV in this disease. The association between β genus HPV and eyelid cutaneous SCC is still uncertain.

## 5. Conclusions

**Figure 4:**
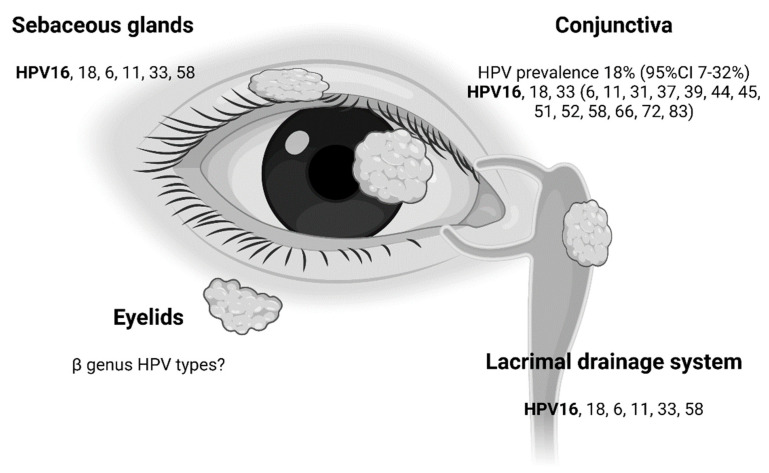
HPV genotypes associated to carcinomas of the ocular adnexa. HPV16 (highlighted in bold) is the most commonly reported genotype in the conjunctiva, lacrimal drainage system and in ocular adnexal sebaceous gland carcinoma. Rarely reported genotypes are shown in parentheses. The figure is created using BioRender.com.

In recent years, more sophisticated molecular techniques, including RNA ISH and high-throughput sequencing, have made it possible to demonstrate the transcription of HPV oncogenes in carcinomas of the ocular adnexa, even in older and formalin-fixed and paraffin-embedded (FFPE) tumor tissue. Today, increasing evidence supports that defined subsets of benign and malignant neoplasia in the ocular adnexa develop in an HPV-dependent pathway. The most frequently involved genotypes in both benign (HPV6, 11) and malignant (HPV16, 18) neoplasia of the ocular adnexa are covered by the L1 prophylactic HPV vaccines and are thereby potentially preventable by vaccination (Figure 4).

HPV status serves as a diagnostic biomarker a subset of head-and-neck carcinomas, and tremendous efforts are now put into the use of HPV status to up and de-escalate the treatment regimens. In the ocular adnexal region, many pieces of the puzzle are still missing in order to incorporate HPV status to the benefit of the patients. To elucidate further the role of HPV in ocular adnexal papillomas and carcinomas, the HPV status, preferentially investigated by the expression of viral oncogenes or a combination of HPV DNA PCR and p16^INK4a^ immunohistochemistry, should be included in future studies of all these tumors.

## Figures and Tables

**Table 1:** An overview of studies (including >5 cases) examining HPV in *conjunctival exophytic papilloma*. HPV; human papillomavirus, PCR; polymerase chain reaction, ISH; in-situ hybridization.

Author, Year	Median Age Years (Range), Gender	HPV+ (DNA PCR)	HPV+ (RNA ISH)	HPV Genotypes	HPV Detection Modality
Mlakar et al., 2015 [9]	49 (28–77), 17M/8F	19/25 (76%)	NA	6, 11	PCR, in-situ hybridization
Takamura et al., 2008 [15]	40 (28–76), 4M/2F	6/6 (100%)	NA	NA	PCR, hybrid capture II
Sjö et al., 2007 [10]	27 (18–65)	86/106 (81%)	NA	6, 11, 45	PCR
Eng et al., 2002 [16]	(9–80), 21M/3F	14/24 (58%)	NA	6, 11	PCR
Nakamura et al., 1997 [17]	51 (20–73), 6M/2F	4/8 (50%)	NA	6	PCR, in-situ hybridization
Saegusa et al., 1995 [11]	38 (14–73), 5M/11F	12/16 (75%)	NA	16	PCR, in-situ hybridization
McDonnell et al., 1987 [18]	25 (1–71)	15/23 (65%)	NA	6	In-situ hybridization

**Table 2:** An overview of studies examining expression of HPV oncogenes in *conjunctival intraepithelial neoplasia and carcinoma*.

Author, Year	Median Age Years (Range)/Gender	HPV+ (DNA)	HPV+ (RNA)	HPV Genotypes	HPV Detection Modality
Griffin et al., 2019 [33]	61 (21–103)/25M, 16F	17/41 (41%)	11/13 (85%) *	16	PCR, RNA ISH, p16^INK4a^
Nagarajan et al., 2019 [37]	62 (36–81)/16M, 15F	NA	8/31 (26%)	High-risk genotypes	RNA ISH
Ramberg et al., 2019 [38]	65 (30–97)/81M, 31F	24/112 (21%)	18/19 (95%) **	6, 11, 16, 33, 39	PCR, RNA ISH, p16^INK4a^
Scott et al., 2002 [39]	NA	10/10 (100%)	10/10 (100%)	16, 18	RT-PCR, DNA ISH

* 13 out of 17 HPV DNA positive cases tested with RNA ISH. ** 19 out of 24 HPV DNA positive cases tested with RNA ISH. HPV; human papillomavirus, PCR; polymerase chain reaction, ISH; in-situ hybridization.

**Table 3:** An overview of studies examining HPV in *papillomas of the lacrimal drainage system*.

Author, Year	Median Age Years (Range)/Gender	HPV+ (DNA PCR)	HPV+ (RNA ISH)	HPV Genotypes	HPV Detection Modality
Jones et al., 2020 [67]	-	3/10 (30%)	NA	6, 11, 16	PCR
Madreperla et al., 1993 [68]	38 (36–54)/3M	2/2 (100%)	NA	11	PCR, DNA ISH
Sjö et al., 2007 [66]	37 (30–56)	4/4 (100%)	2/2 (100%)	6, 11	PCR, DNA ISH, RNA ISH
Vickers et al., 2010 [69]	53/F	1/1 (100%)	NA	11	PCR
Nakamura et al., 1997 [17]	38 (26–50)/1F, 1M	1/2 (50%)	NA	16	DNA ISH, PCR
Buchwald et al., 1996 [70]	-	1/1 (100%)	NA	6/11	DNA ISH

PCR; Polymerase chain reaction, ISH; in-situ hybridization.

**Table 4:** An overview of studies examining HPV in *carcinomas of the lacrimal drainage system*.

Author, Year	Median Age Years (Range)/Gender	HPV+ (DNA PCR)	HPV+ (RNA ISH)	HPV Genotypes	HPV Detection Modality
Afrogheh et al., 2016 [42]	60 (34–75)/4M, 5F	8/9 (89%)	NA	16, 33, 58	DNA ISH, PCR, p16^INK4a^
Madreperla et al., 1993 [68]	-	1/2 (50%) *	NA	18	DNA ISH, PCR
Sjö et al., 2007 [66]	61 (33–86)/4M, 2F	4/6 (67%)	0/4 (0%)	6, 11, 16 **	PCR, DNA ISH, RNA ISH
Jones et al., 2020 [67]	-	2/4 (50%)	NA	16	PCR

HPV; human papillomavirus, PCR; Polymerase chain reaction, ISH; in-situ hybridization, NA; not applicable. * Only two carcinomas were available to PCR. ** Co-cominant infection with low-risk 6 or 11 and high-risk HPV16.

**Table 5:** An overview of studies examining HPV in *sebaceous gland carcinomas of the ocular adnexa*.

Author, Year	Median Age Years (Range)/Gender	HPV+ (DNA PCR)	HPV+ (RNA ISH)	HPV Genotypes	HPV Detection Modality
Hayashi et al., 1994 [72]	63 (52–83)/6M:7F	13/21 (62%)	NA	16, 18, 31, 33, 6, 11	DNA ISH
Gonzalez-Fernandez et al., 1998 [75]	72 (32–90)/7F	0/7 (0%)	NA	NA	DNA ISH, PCR
Kwon et al., 2015 [74]	72 (45–86)/4M:10F	0/14 (0%)	NA	NA	HPV chip test
Liau et al., 2014 [79]	NA/8M:16F	1/24 (4%)	NA	16	PCR
Stagner et al., 2016 [2]	NA	1/24 (4%)	0/18 (0%)	16	PCR, RNA ISH
Tetzlaff et al., 2019 [77]	68 (44–93)/13M:16F	4/29 (14%)	4/29 (14%)	16, 18	RNA ISH, RNA sequencing
Chauhan et al., 2019 [76]	mean 56.8±13.9 (25–88)/16M:14F	0/30 (0%)	NA	NA	PCR
Moore et al., 2021 [78]	mean 73 (27–98)/8M:10F	NA	2/11 (18%)	High-risk HPV	RNA ISH

HPV; Human papillomavirus, PCR; Polymerase chain reaction, ISH; in-situ hybridization, NA; not applicable.

## References

[1] M’Fadyean J., Hobday F. (1898). Note on the Experimental Transmission of Warts in the Dog. J. Comp. Pathol..

[2] Stagner A.M., Afrogheh A.H., Jakobiec F.A., Iacob C.E., Grossniklaus H.E., Deshpande V., Maske C., Hiss D.C., Faquin W.C. (2016). p16 Expression Is Not a Surrogate Marker for High-Risk Human Papillomavirus Infection in Periocular Sebaceous Carcinoma. Am. J. Ophthalmol..

[3] Lass J.H., Jenson A.B., Papale J.J., Albert D.M. (1983). Papillomavirus in human conjunctival papillomas. Am. J. Ophthalmol..

[4] McDonnell J.M., McDonnell P.J., Mounts P., Wu T.C., Green W.R. (1986). Demonstration of papillomavirus capsid antigen in human conjunctival neoplasia. Arch. Ophthalmol..

[5] Sjö N., Heegaard S., Prause J.U. (2000). Conjunctival papilloma. A histopathologically based retrospective study. Acta Ophthalmol. Scand..

[6] Kaliki S., Arepalli S., Shields C.L., Klein K., Sun H., Hysenj E., Lally S.E., Shields J.A. (2013). Conjunctival papilloma: Features and outcomes based on age at initial examination. JAMA Ophthalmol..

[7] Furdova A., Stopkova A., Kapitanova K., Kobzova D., Babal P. (2018). Conjuctival lesions—the relationship of papillomas and squamous cell carcinoma to HPV infection. Cesk. Slov. Oftalmol..

[8] Theotoka D., Morkin M.I., Galor A., Karp C.L. (2019). Update on Diagnosis and Management of Conjunctival Papilloma. Eye Vis..

[9] Mlakar J., Kocjan B.J., Hosnjak L., Pizem J., Beltram M., Gale N., Drnovsek-Olup B., Poljak M. (2015). Morphological characteristics of conjunctival squamous papillomas in relation to human papillomavirus infection. Br. J. Ophthalmol..

[10] Sjö N.C., von Buchwald C., Cassonnet P., Norrild B., Prause J.U., Vinding T., Heegaard S. (2007). Human papillomavirus in normal conjunctival tissue and in conjunctival papilloma: Types and frequencies in a large series. Br. J. Ophthalmol..

[11] Saegusa M., Takano Y., Hashimura M., Okayasu I., Shiga J. (1995). HPV type 16 in conjunctival and junctional papilloma, dysplasia, and squamous cell carcinoma. J. Clin. Pathol..

[12] Benevides Dos Santos P.J., Borborema Dos Santos C.M., Mendonça R.R., Vieira Do Carmo M.A., Astofi-Filho S. (2005). Human papillomavirus type 13 infecting the conjunctiva. Diagn. Microbiol. Infect. Dis..

[13] Buggage R.R., Smith J.A., Shen D., Chan C.C. (2002). Conjunctival papillomas caused by human papillomavirus type 33. Arch. Ophthalmol..

[14] Sjö N.C., Heegaard S., Prause J.U., Von Buchwald C., Lindeberg H. (2001). Human papillomavirus in conjunctival papilloma. Br. J. Ophthalmol..

[15] Takamura Y., Kubo E., Tsuzuki S., Akagi Y. (2008). Detection of human papillomavirus in pterygium and conjunctival papilloma by hybrid capture II and PCR assays. Eye.

[16] Eng H.L., Lin T.M., Chen S.Y., Wu S.M., Chen W.J. (2002). Failure to detect human papillomavirus DNA in malignant epithelial neoplasms of conjunctiva by polymerase chain reaction. Am. J. Clin. Pathol..

[17] Nakamura Y., Mashima Y., Kameyama K., Mukai M., Oguchi Y. (1997). Detection of human papillomavirus infection in squamous tumours of the conjunctiva and lacrimal sac by immunohistochemistry, in situ hybridisation, and polymerase chain reaction. Br. J. Ophthalmol..

[18] McDonnell P.J., McDonnell J.M., Kessis T., Green W.R., Shah K.V. (1987). Detection of human papillomavirus type 6/11 DNA in conjunctival papillomas by in situ hybridization with radioactive probes. Hum. Pathol..

[19] Ramberg I., Sjo N.C., Bonde J.H., Heegaard S. (2019). Inverted papilloma of the conjunctiva. BMJ Open Ophthalmol..

[20] Bata B.M., Salvi S.M., Mudhar H.S. (2021). Conjunctival invasive squamous carcinoma arising from a dysplastic inverted papilloma, both positive for HPV16. Can. J. Ophthalmol..

[21] Heuring A.H., Hutz W.W., Eckhardt H.B., Bohle R.M. (1998). Invertiertes Transitionalzellpapillom der Bindehaut mit peripherer karzinomatöser Entartung. Klin. Mon. Augenheilkd..

[22] Lassalle S., Maschi C., Caujolle J.P., Giordanengo V., Hofman P. (2017). Inverted conjunctival papilloma: A certainly underestimated high-risk lesion for carcinomatous transformation-a case report. Can. J. Ophthalmol..

[23] Zhao R.W., Guo Z.Q., Zhang R.X. (2016). Human papillomavirus infection and the malignant transformation of sinonasal inverted papilloma: A meta-analysis. J. Clin. Virol..

[24] Cervantes G., Rodríguez A.A., Leal A.G. (2002). Squamous cell carcinoma of the conjunctiva: Clinicopathological features in 287 cases. Can. J. Ophthalmol..

[25] Tunc M., Char D.H., Crawford B., Miller T. (1999). Intraepithelial and invasive squamous cell carcinoma of the conjunctiva: Analysis of 60 cases. Br. J. Ophthalmol..

[26] Merz L.E., Afriyie O., Jiagge E., Adjei E., Foltin S.K., Ludwig M.L., McHugh J.B., Brenner J.C., Merajver S.D. (2019). Clinical characteristics, HIV status, and molecular biomarkers in squamous cell carcinoma of the conjunctiva in Ghana. Health Sci. Rep..

[27] Karcioglu Z.A., Toth J. (2000). Relation between p53 overexpression and clinical behavior of ocular/orbital invasion of conjunctival squamous cell carcinoma. Ophthalmic Plast. Reconstr. Surg..

[28] Muchengeti M., Bohlius J., Dhokotera T.G. (2021). Conjunctival cancer in people living with HIV. Curr. Opin. Infect. Dis..

[29] Gichuhi S., Macharia E., Kabiru J., Zindamoyen A.M., Rono H., Ollando E., Wachira J., Munene R., Maina J., Onyuma T. (2016). Topical fluorouracil after surgery for ocular surface squamous neoplasia in Kenya: A randomised, double-blind, placebo-controlled trial. Lancet Glob. Health.

[30] Tabin G., Levin S., Snibson G., Loughnan M., Taylor H. (1997). Late recurrences and the necessity for long-term follow-up in corneal and conjunctival intraepithelial neoplasia. Ophthalmology.

[31] Siedlecki A.N., Tapp S., Tosteson A.N., Larson R.J., Karp C.L., Lietman T., Zegans M.E. (2016). Surgery Versus Interferon Alpha-2b Treatment Strategies for Ocular Surface Squamous Neoplasia: A Literature-Based Decision Analysis. Cornea.

[32] Giannaccare G., Bernabei F., Angi M., Pellegrini M., Maestri A., Romano V., Scorcia V., Rothschild P.R. (2021). Iatrogenic Ocular Surface Diseases Occurring during and/or after Different Treatments for Ocular Tumours. Cancers.

[33] Griffin H., Mudhar H.S., Rundle P., Shiraz A., Mahmood R., Egawa N., Quint W., Rennie I.G., Doorbar J. (2019). Human papillomavirus type 16 causes a defined subset of conjunctival in situ squamous cell carcinomas. Mod. Pathol..

[34] Hirst L.W. (2007). Randomized controlled trial of topical mitomycin C for ocular surface squamous neoplasia: Early resolution. Ophthalmology.

[35] Ramberg I., Møller-Hansen M., Toft P.B., Funding M., Heegaard S. (2020). Human papillomavirus infection plays a role in conjunctival squamous cell carcinoma: A systematic review and meta-analysis of observational studies. Acta Ophthalmol..

[36] Carreira H., Coutinho F., Carrilho C., Lunet N. (2013). HIV and HPV infections and ocular surface squamous neoplasia: Systematic review and meta-analysis. Br. J. Cancer.

[37] Nagarajan P., El-Hadad C., Gruschkus S.K., Ning J., Hudgens C.W., Sagiv O., Gross N., Tetzlaff M.T., Esmaeli B. (2019). PD-L1/PD1 Expression, Composition of Tumor-Associated Immune Infiltrate, and HPV Status in Conjunctival Squamous Cell Carcinoma. Investig. Ophthalmol. Vis. Sci..

[38] Ramberg I., Toft P.B., Georgsen J.B., Siersma V.D., Funding M., Jensen D.H., Von Buchwald C., Heegaard S. (2019). Conjunctival intraepithelial neoplasia and carcinoma: Distinct clinical and histological features in relation to human papilloma virus status. Br. J. Ophthalmol..

[39] Scott I.U., Karp C.L., Nuovo G.J. (2002). Human papillomavirus 16 and 18 expression in conjunctival intraepithelial neoplasia. Ophthalmology.

[40] Egawa N., Egawa K., Griffin H., Doorbar J. (2015). Human Papillomaviruses; Epithelial Tropisms, and the Development of Neoplasia. Viruses.

[41] Ateenyi-Agaba C., Franceschi S., Wabwire-Mangen F., Arslan A., Othieno E., Binta-Kahwa J., van Doorn L.J., Kleter B., Quint W., Weiderpass E. (2010). Human papillomavirus infection and squamous cell carcinoma of the conjunctiva. Br. J. Cancer.

[42] Afrogheh A., Jakobiec F., Hammon R., Grossniklaus H., Rocco J., Lindeman N., Sadow P., Faquin W. (2016). Evaluation for high-risk HPV in squamous cell carcinomas and precursor lesions arising in the conjunctiva and lacrimal sac. Am. J. Surg. Pathol..

[43] Chauhan S., Sen S., Sharma A., Kashyap S., Tandon R., Bajaj M.S., Pushker N., Vanathi M., Chauhan S.S. (2018). p16(INK4a) overexpression as a predictor of survival in ocular surface squamous neoplasia. Br. J. Ophthalmol..

[44] Tabrizi S.N., McCurrach F.E., Drewe R.H., Borg A.J., Garland S.M., Taylor H.R. (1997). Human papillomavirus in corneal and conjunctival carcinoma. Aust. N. Z. J. Ophthalmol..

[45] Woods M., Chow S., Heng B., Glenn W., Whitaker N., Waring D., Iwasenko J., Rawlinson W., Coroneo M.T., Wakefield D. (2013). Detecting human papillomavirus in ocular surface diseases. Investig. Ophthalmol. Vis. Sci..

[46] Sen S., Sharma A., Panda A. (2007). Immunohistochemical localization of human papilloma virus in conjunctival neoplasias: A retrospective study. Indian J. Ophthalmol..

[47] Auw-Haedrich C., Martin G., Spelsberg H., Sundmacher R., Freudenberg N., Maier P., Reinhard T. (2008). Expression of p16 in conjunctival intraepithelial neoplasia does not correlate with HPV-infection. Open Ophthalmol. J..

[48] Chauhan S., Sen S., Sharma A., Dar L., Kashyap S., Kumar P., Bajaj M.S., Tandon R. (2012). Human papillomavirus: A predictor of better survival in ocular surface squamous neoplasia patients. Br. J. Ophthalmol..

[49] Jung S.M., Lin H.C., Chu P.H., Wu H.H., Shiu T.F., Shang L.H., Lai C.H. (2006). Expression of cell cycle-regulatory proteins, MIB-1, p16, p53, and p63, in squamous cell carcinoma of conjunctiva: Not associated with human papillomavirus infection. Virchows Arch..

[50] Kuo K.T., Chang H.C., Hsiao C.H., Lin M.C. (2006). Increased Ki-67 proliferative index and absence of P16INK4 in CIN-HPV related pathogenic pathways different from cervical squamous intraepithelial lesion. Br. J. Ophthalmol..

[51] Shrestha T., Choi W., Kim G.E., Yang J.M., Yoon K.C. (2019). Human papilloma virus identification in ocular surface squamous neoplasia by p16 immunohistochemistry and DNA chip test: A strobe-compliant article. Medicine.

[52] Moyer A.B., Roberts J., Olsen R.J., Chevez-Barrios P. (2018). Human papillomavirus-driven squamous lesions: High-risk genotype found in conjunctival papillomas, dysplasia, and carcinoma. Am. J. Dermatopathol..

[53] Naghashfar Z., McDonnell P.J., McDonnell J.M., Green W.R., Shah K.V. (1986). Genital tract papillomavirus type 6 in recurrent conjunctival papilloma. Arch. Ophthalmol..

[54] Egbert J.E., Kersten R.C. (1997). Female genital tract papillomavirus in conjunctival papillomas of infancy. Am. J. Ophthalmol..

[55] Minchiotti S., Masucci L., Serapiao Dos Santos M., Perrella E., Graffeo R., Lambiase A., Bonini S. (2006). Conjunctival papilloma and human papillomavirus: Identification of HPV types by PCR. Eur. J. Ophthalmol..

[56] Trottier H., Mayrand M.H., Coutlée F., Monnier P., Laporte L., Niyibizi J., Carceller A.M., Fraser W.D., Brassard P., Lacroix J. (2016). Human papillomavirus (HPV) perinatal transmission and risk of HPV persistence among children: Design, methods and preliminary results of the HERITAGE study. Papillomavirus Res..

[57] Sonnex C., Strauss S., Gray J.J. (1999). Detection of human papillomavirus DNA on the fingers of patients with genital warts. Sex. Transm. Dis..

[58] Iovieno A., Piana S., Chiesi L., Fodero C., Fontana L. (2018). Human papillomavirus (HPV)-associated trilateral squamous neoplasia in immunocompetent individual. Int. Ophthalmol..

[59] Gichuhi S., Sagoo M.S., Weiss H.A., Burton M.J. (2013). Epidemiology of ocular surface squamous neoplasia in Africa. Trop. Med. Int. Health.

[60] McGrath L.A., Salvi S.M., Sandramouli S., Bhatt R., Cuschieri K., Mudhar H.S. (2018). Squamous cell carcinoma in the anophthalmic socket: A series of four cases with HPV-16 profiling. Br. J. Ophthalmol..

[61] Ramberg I., Toft P.B., Heegaard S. (2020). Carcinomas of the lacrimal drainage system. Surv. Ophthalmol..

[62] Kroll J., Busse H. (2008). Tumoren der ableitenden tranenwege. Klin. Mon. Augenheilkd..

[63] Ryan S.J., Font R.L. (1973). Primary epithelial neoplasms of the lacrimal sac. Am. J. Ophthalmol..

[64] Stefanyszyn M.A., Hidayat A.A., Pe’er J.J., Flanagan J.C. (1994). Lacrimal sac tumors. Ophthalmic Plast. Reconstr. Surg..

[65] Anderson K.K., Lessner A.M., Hood I., Mendenhall W., Stringer S., Warren R. (1994). Invasive transitional cell carcinoma of the lacrimal sac arising in an inverted papilloma. Arch. Ophthalmol..

[66] Sjö N.C., von Buchwald C., Cassonnet P., Flamant P., Heegaard S., Norrild B., Prause J.U., Orth G. (2007). Human papillomavirus: Cause of epithelial lacrimal sac neoplasia?. Acta Ophthalmol..

[67] Jones H., Gane S., Rimmer J., Cuschieri K., Lund V.J. (2020). HPV may not play a role in all lacrimal transitional cell papilloma. Rhinology.

[68] Madreperla S.A., Green W.R., Daniel R., Shah K.V. (1993). Human papillomavirus in primary epithelial tumors of the lacrimal sac. Ophthalmology.

[69] Vickers J.L., Matherne R.J., Allison A.W., Wilkerson M.G., Tyring S.K., Bartlett B.L., Rady P.L., Kelly B.C. (2010). Transitional cell neoplasm of the nasolacrimal duct associated with human papillomavirus type 11. J. Cutan. Pathol..

[70] Buchwald C., Skoedt V., Tos M. (1996). An expansive papilloma of the nasolachrymal drainage system harbouring human papilloma virus. Rhinology.

[71] Alam M.S., Mukherjee B., Krishnakumar S. (2021). Clinical profile and management outcomes of lacrimal drainage system malignancies. Orbit.

[72] Hayashi N., Furihata M., Ohtsuki Y., Ueno H. (1994). Search for accumulation of p53 protein and detection of human papillomavirus genomes in sebaceous gland carcinoma of the eyelid. Virchows Arch..

[73] North J.P., Golovato J., Vaske C.J., Sanborn J.Z., Nguyen A., Wu W., Goode B., Stevers M., McMullen K., Perez White B.E. (2018). Cell of origin and mutation pattern define three clinically distinct classes of sebaceous carcinoma. Nat. Commun..

[74] Kwon M.J., Shin H.S., Nam E.S., Cho S.J., Lee M.J., Lee S., Park H.R. (2015). Comparison of HER2 gene amplification and KRAS alteration in eyelid sebaceous carcinomas with that in other eyelid tumors. Pathol. Res. Pract..

[75] Gonzalez-Fernandez F., Kaltreider S.A., Patnaik B.D., Retief J.D., Bao Y., Newman S., Stoler M.H., Levine P.A. (1998). Sebaceous carcinoma. Tumor progression through mutational inactivation of p53. Ophthalmology.

[76] Chauhan S., Sen S., Singh N., Sharma A., Pushker N., Kashyap S., Chawla B. (2019). Human papillomavirus in ocular malignant tumours: A study from a tertiary eye care centre in North India. Can. J. Ophthalmol..

[77] Tetzlaff M.T., Curry J.L., Ning J., Sagiv O., Kandl T., Peng B., Bell D., Routbort M.J., Hudgens C.W., Ivan D. (2018). Distinct biological types of ocular adnexal sebaceous carcinoma: HPV-driven and virus-negative tumors arise through non-overlapping molecular-genetic alterations. Clin. Cancer Res..

[78] Moore R.F., Zhang X.R., Allison D.B., Rooper L.M., Campbell A.A., Eberhart C.G. (2021). High-risk human papillomavirus and ZEB1 in ocular adnexal sebaceous carcinoma. J. Cutan. Pathol..

[79] Liau J.Y., Liao S.L., Hsiao C.H., Lin M.C., Chang H.C., Kuo K.T. (2014). Hypermethylation of the CDKN2A gene promoter is a frequent epigenetic change in periocular sebaceous carcinoma and is associated with younger patient age. Hum. Pathol..

[80] Reifler D.M., Hornblass A. (1986). Squamous cell carcinoma of the eyelid. Surv. Ophthalmol..

[81] Cook B.E., Bartley G.B. (1999). Epidemiologic characteristics and clinical course of patients with malignant eyelid tumors in an incidence cohort in Olmsted County, Minnesota. Ophthalmology.

[82] Arroyo Mühr L.S., Hultin E., Dillner J. (2021). Transcription of Human Papillomaviruses in Non-Melanoma Skin Cancers of the Immunosuppressed. Int J. Cancer.

[83] Ramezani M., Baharzadeh F., Almasi A., Sadeghi M. (2020). A Systematic Review and Meta-Analysis: Evaluation of the β-Human Papillomavirus in Immunosuppressed Individuals with Cutaneous Squamous Cell Carcinoma. BioMedicine.

[84] Rollison D.E., Viarisio D., Amorrortu R.P., Gheit T., Tommasino M. (2019). An Emerging Issue in Oncogenic Virology: The Role of Beta Human Papillomavirus Types in the Development of Cutaneous Squamous Cell Carcinoma. J. Virol..

[85] Doorbar J., Egawa N., Griffin H., Kranjec C., Murakami I. (2015). Human papillomavirus molecular biology and disease association. Rev. Med. Virol..

